# Correction to: Methods and participant characteristics in the Cancer risk in vegetarians consortium: a cross-sectional analysis across 11 prospective studies

**DOI:** 10.1186/s12889-025-22844-8

**Published:** 2025-06-11

**Authors:** Yashvee Dunneram, Jia Yi Lee, Cody Z. Watling, Gary E. Fraser, Fayth Miles, Dorairaj Prabhakaran, Krithiga Shridhar, Dimple Kondal, Viswanathan Mohan, Mohammed K. Ali, Kabayam M. Venkat Narayan, Nikhil Tandon, Tammy Y. N. Tong, Tina H. T. Chiu, Ming-Nan Lin, Chin-Lon Lin, Hsin-Chou Yang, Yu-Jen Liang, Darren C. Greenwood, Huaidong Du, Zhengming Chen, Canqing Yu, Maria G. Kakkoura, Gillian K. Reeves, Keren Papier, Sarah Floud, Rashmi Sinha, Linda M. Liao, Erikka Loftfield, Janet E. Cade, Timothy J. Key, Aurora Perez-Cornago

**Affiliations:** 1 Cancer Epidemiology Unit,Nuffield, Department of Population Health, University of Oxford, Oxford, OX3 7LF UK; 2 Human Nutrition and Exercise Research Centre, Faculty of Medical Sciences, Newcastle University, Newcastle Upon Tyne, NE2 4HH UK; 3 Centre for Nutrition, Healthy Lifestyle, and Disease Prevention, School of Public Health, Loma Linda University, Loma Linda, CA USA; 4 Centre for Chronic Disease Control, New Delhi, India; 5 Public Health Foundation of India, Gurugram, Haryana India; 6 Emory Global Diabetes Research Center, Woodruff Health Sciences Centerand, Emory University, Atlanta, GA USA; 7 London School of Hygiene and Tropical Medicine, London, UK; 8 Centre for Health Analytics, Trivedi School of Bioscience, Ashoka University, Research, and Trends, Sonipat, Haryana India; 9 Madras Diabetes Research Foundation (ICMR Center for Advanced Research On Diabetes) and Dr. Mohan’s Diabetes Specialities Centre, Chennai, India; 10 Hubert Department of Global Health, Emory University, Atlanta, GA USA; 11 Department of Epidemiology, Rollins School of Public Health, Emory University, Atlanta, GA USA; 12 Department of Family and Preventive Medicine, School of Medicine, Emory University, Atlanta, GA USA; 13 Department of Endocrinology, All India Institute of Medical Sciences, New Delhi, India; 14 Department of Nutritional Science, Fu-Jen Catholic University, New Taipei City, Taiwan; 15 Department of Family Medicine, Dalin Tzu Chi Hospital, Buddhist Tzu Chi Medical Foundation, Chiayi, 622 Taiwan; 16 Department of Family Medicine, College of Medicine, Tzu Chi University, Hualien, 970 Taiwan; 17 Buddhist Tzu Chi Medical Foundation, Hualien City, Taiwan; 18 Institute of Statistical Science, Academia Sinica, Taipei City, Taiwan; 19 School of Medicine, University of Leeds, Leeds, UK; 20 Clinical Trial Service Unit & Epidemiological Studies Unit, Nuffield, Department of Population Health, University of Oxford, Oxford, UK; 21 Medical Research Council Population Health Research Unit, University of Oxford, Oxford, UK; 22 Department of Epidemiology & Biostatistics, School of Public Health, Peking University, Beijing, China; 23 Center for Public Health and Epidemic Preparedness and Response, Peking University, Beijing, China; 24 Division of Cancer Epidemiology and Genetics, National Cancer Institute, Bethesda, MD USA; 25 Nutritional Epidemiology Group, School of Food Science and Nutrition, University of Leeds, Leeds, UK

**Correction to: BMC Public Health 24, 2095 (2024)**

10.1186/s12889-024-19209-y

Following publication of the original article [1], the authors identified an error in Table 6 and Table S5. The percentage for the every used oral contraceptives value for the NIH-AARP study has been corrected to 39%.

**Table 6:** Women-specific characteristics by cohort (n = 1,546,217)^1^

Cohort	Age at menarche ≤ 12 years	Parous	Age at first birth ≥ 25 years	Postmenopausal	Age at menopause ≥ 50 years^2^	Ever used oral contraceptive	Ever used HRT
*Cohorts with large proportions of vegetarians*							
Adventist Health Study-2	51.4	83.1	31.7	77.0	64.5	58.8	39.4
CARRS-1	12.2	94.1	-	36.9	19.7	3.5	-
CARRS-2	12.9	93.8	-	41.9	23.4	6.9	
EPIC-Oxford	39.8	61.0	38.2	40.2	54.8	73.7	18.2
Oxford Vegetarian Study	-	47.5	29.4	26.6	-	55.3	-
Tzu Chi Health Study	8.6	91.8	-	63.0	54.2	15.7	19.6
UK Women’s Cohort Study	41.6	77.2	44.8	60.9	48.0	66.9	27.2
***Very large cohorts***							
China Kadoorie Biobank	5.5	98.7	32.5	57.2	43.1	9.8	-
Million Women Study	38.8	88.4	36.4	100	56.8	61.3	53.4
NIH-AARP	48.6	83.7	23.5	100	39.6	39.0	52.9
UK Biobank	37.6	81.2	47.1	77.8	63.3	81.1	37.7
**All cohorts combined**	33.5	87.3	35.3	84.2	52.1	50.6	37.8

Abbreviations: CARRS, Centre for cArdiometabolic Risk Reduction in South Asia; HRT, hormone replacement therapy; EPIC, European Prospective Investigation into Cancer and Nutrition; NIH-AARP, National Institutes of Health-AARP Diet and Health Study.

^1^Values are % of women within cohort.

^2^Postmenopausal women only.

“-” indicates that no information was available for this variable in the specified cohort.

**Table S5:** Baseline characteristics of women by cohort^1^

	Total	AHS-2	CARRS-1	CARRS-2	EPIC-Oxford	OVS	TCHS	UKWCS	CKB	MWS	NIH-AARP	UK Biobank
N	1,546,217	42,194	6,372	5,065	41,335	6,480	3,329	30,148	300,909	639,026	215,905	255,454
Living with partner												
Yes	1,145,967 (74.1)	27,503 (65.2)	5,671 (89.0)	4,332 (85.5)	27,614 (66.8)	3,497 (54.0)	2824 (84.8)	22,282 (73.9)	267,799 (89.0)	511,789 (80.1)	95,749 (44.3)	176,907 (69.3)
No	336,711 (21.8)	13,937 (33.0)	701 (11.0)	733 (14.5)	13,649 (33.0)	2,934 (45.3)	504 (15.1)	7,415 (24.6)	33,110 (11.0)	118,337 (18.5)	118,140 (54.7)	27,251 (10.7)
Unknown	63,539 (4.1)	754 (1.8)	0 (0.00)	0 (0.00)	72 (0.17)	49 (0.76)	1 (0.00)	451 (1.5)	0 (0.00)	8,900 (1.4)	2,016 (0.93)	51,296 (20.1)
Educational status												
Less than secondary/high school	514,795 (33.3)	3,052 (7.2)	1,568 (24.6)	1,271 (25.1)	5,426 (13.1)	309 (4.8)	1023 (30.7)	4,671 (15.5)	170,715 (56.7)	217,050 (34.0)	67,342 (31.2)	42,368 (16.6)
Secondary/high school or equivalent	572,012 (37.0)	18,125 (43.0)	3,859 (60.6)	2,908 (57.4)	16,422 (39.7)	4,573 (70.6)	1636 (49.1)	15,539 (51.5)	116,872 (38.8)	305,150 (47.8)	22,927 (10.6)	64,001 (25.1)
University degree or equivalent	430,226 (27.8)	20,431 (48.4)	945 (14.8)	884 (17.5)	16,406 (39.7)	434 (6.7)	670 (20.1)	7,372 (24.5)	13,322 (4.4)	106,496 (16.7)	118,637 (54.9)	144,629 (56.6)
Unknown	29,184 (1.9)	586 (1.4)	0 (0.00)	2 (0.04)	3,081 (7.5)	1,164 (18.0)	0 (0.00)	2,566 (8.5)	0 (0.00)	10,330 (1.6)	6,999 (3.2)	4,456 (1.7)
Cigarette smoking												
Never	974,560 (63.0)	35,304 (83.7)	6,094 (95.6)	4,985 (98.4)	25,457 (61.6)	3,595 (55.5)	3274 (98.3)	16,851 (55.9)	285,673 (94.9)	346,928 (54.3)	94,000 (43.5)	152,399 (59.7)
Previous	406,326 (26.3)	6,166 (14.6)	5 (0.08)	21 (0.41)	11,253 (27.2)	1,809 (27.9)	40 (1.20)	9,148 (30.3)	2,619 (0.87)	211,953 (33.2)	84,058 (38.9)	79,254 (31.0)
Current	146,379 (9.5)	410 (0.97)	58 (0.91)	59 (1.2)	4,439 (10.7)	1,049 (16.2)	15 (0.45)	3,224 (10.7)	12,617 (4.2)	71,277 (11.2)	30,343 (14.1)	22,888 (9.0)
Unknown	18,952 (1.2)	314 (0.74)	215 (3.4)	0 (0.00)	186 (0.45)	27 (0.42)	0 (0.00)	925 (3.1)	0 (0.00)	8,868 (1.4)	7,504 (3.5)	913 (0.36)
Physical activity												
Inactive	357,643 (23.1)	8,553 (20.3)	2,681 (42.1)	3,815 (75.3)	23,253 (56.3)	2,596 (40.1)	1248 (37.5)	22,033 (73.1)	99,953 (33.2)^2^	36,813 (5.8)	79,280 (36.7)	77,418 (30.3)
Moderately active	426,785 (27.6)	13,711 (32.5)	797 (12.5)	491 (9.7)	6,931 (16.8)	1,871 (28.9)	1082 (32.5)	7,074 (23.5)	100,654 (33.4)^2^	70,898 (11.1)	99,164 (45.9)	124,112 (48.6)
Highly active	570,911 (36.9)	17,753 (42.1)	1,494 (23.4)	40 (0.79)	4,770 (11.5)	1,831 (28.3)	999 (30.0)	1,032 (3.4)	100,302 (33.3)^2^	364,921 (57.1)	34,746 (16.1)	43,023 (16.8)
Unknown	190,878 (12.3)	2,177 (5.2)	1,400 (22.0)	719 (14.2)	6,381 (15.4)	182 (2.8)	0 (0.00)	9 (0.03)	0 (0.00)	166,394 (26.0)	2,715 (1.3)	10,901 (4.3)
History of diabetes												
Yes	71,748 (4.6)	3,195 (7.6)	777 (12.2)	830 (16.4)	459 (1.1)	33 (0.51)	155 (4.7)	544 (1.8)	18,426 (6.1)	21,570 (3.4)	16,204 (7.5)	9,555 (3.7)
No	1,466,761 (94.9)	38,512 (91.3)	5,569 (87.4)	4,231 (83.5)	36,684 (88.7)	6,419 (99.1)	3174 (95.3)	27,293 (90.5)	282,483 (93.9)	617,456 (96.6)	199,701 (92.5)	245,239 (96.0)
Unknown	7,708 (0.50)	487 (1.2)	26 (0.41)	4 (0.08)	4,192 (10.1)	28 (0.43)	0 (0.00)	2,311 (7.7)	0 (0.00)	0 (0.00)	0 (0.00)	660 (0.26)
Age at menarche												
≤ 10 years	60,622 (3.9)	3,658 (8.7)	16 (0.25)	18 (0.36)	1,800 (4.4)	-	3 (0.09)	1,760 (5.8)	278 (0.09)	26,924 (4.2)	14,720 (6.8)	11,445 (4.5)
11–12 years	457,022 (29.6)	18,046 (42.8)	759 (11.9)	635 (12.5)	14,635 (35.4)	-	282 (8.47)	10,787 (35.8)	16,130 (5.4)	220,840 (34.6)	90,177 (41.8)	84,731 (33.2)
13–14 years	624,908 (40.4)	15,807 (37.5)	3,931 (61.7)	2,817 (55.6)	18,925 (45.8)	-	1482 (44.5)	13,111 (43.5)	84,506 (28.1)	285,866 (44.7)	89,002 (41.2)	109,461 (42.8)
≥ 15 years	375,784 (24.3)	4,365 (10.3)	1,664 (26.1)	1,570 (31.0)	5,399 (13.1)	-	1536 (46.1)	3,877 (12.9)	199,947 (66.4)	95,559 (15.0)	19,860 (9.2)	42,007 (16.4)
Unknown	27,881 (1.8)	318 (0.75)	2 (0.03)	25 (0.49)	576 (1.4)	6,480 (100)	26 (0.8)	613 (2.0)	48 (0.02)	9,837 (1.5)	2,146 (0.99)	7,810 (3.1)
Parity												
None	187,425 (12.1)	6,542 (15.5)	329 (5.2)	295 (5.8)	15,748 (38.1)	3,244 (50.1)	255 (7.66)	3,739 (12.4)	3,956 (1.3)	72,831 (11.4)	32,816 (15.2)	47,670 (18.7)
One	254,901 (16.5)	5,457 (12.9)	546 (8.6)	385 (7.6)	5,357 (13.0)	917 (14.2)	184 (5.53)	3,748 (12.4)	103,690 (34.5)	78,095 (12.2)	22,460 (10.4)	34,062 (13.3)
Two	593,107 (38.4)	13,342 (31.6)	1,692 (26.6)	1,287 (25.4)	12,261 (29.7)	1,386 (21.4)	1148 (34.5)	11,668 (38.7)	95,596 (31.8)	287,141 (44.9)	55,974 (25.9)	111,612 (43.7)
3–4	430,594 (27.8)	13,045 (30.9)	2,489 (39.1)	1,983 (39.2)	7,062 (17.1)	714 (11.0)	1550 (46.6)	7,255 (24.1)	77,933 (25.9)	182,369 (28.5)	78,618 (36.4)	57,576 (22.5)
5–9	70,558 (4.6)	3,066 (7.3)	1,211 (19.0)	1,042 (20.6)	526 (1.3)	63 (0.97)	173 (5.20)	603 (2.0)	19,542 (6.5)	17,126 (2.7)	23,014 (10.7)	4,192 (1.6)
≥ 10	1,305 (0.08)	133 (0.32)	59 (0.93)	52 (1.0)	2 (0.00)	1 (0.02)	0 (0.00)	12 (0.04)	192 (0.06)	63 (0.01)	746 (0.35)	45 (0.02)
Unknown	8,327 (0.54)	609 (1.4)	46 (0.72)	21 (0.41)	379 (0.92)	155 (2.4)	19 (0.6)	3,123 (10.4)	0 (0.00)	1,401 (0.22)	2,277 (1.1)	297 (0.12)
Age at first birth												
≤ 19 years	157,185 (10.2)	5,947 (14.1)	-	-	1,489 (3.6)	195 (3.0)	-	1,661 (5.5)	28,021 (9.3)	62,803 (9.8)	37,301 (17.3)	19,768 (7.7)
20–24 years	619,652 (40.1)	13,002 (30.8)	-	-	7,934 (19.2)	967 (14.9)	-	8,101 (26.9)	170,982 (56.8)	258,825 (40.5)	92,737 (43.0)	67,104 (26.3)
25–29 years	401,728 (26.0)	8,627 (20.4)	-	-	9,889 (23.9)	1,259 (19.4)	-	8,883 (29.5)	86,929 (28.9)	173,713 (27.2)	38,202 (17.7)	74,226 (29.1)
30–34 years	108,882 (7.0)	3,479 (8.2)	-	-	4,444 (10.8)	490 (7.6)	-	3,434 (11.4)	9,222 (3.1)	45,920 (7.2)	9,717 (4.5)	32,176 (12.6)
35–39 years	30,023 (1.9)	1,049 (2.5)	-	-	1,248 (3.0)	134 (2.1)	-	1,025 (3.4)	1,433 (0.48)	11,223 (1.8)	2,387 (1.1)	11,524 (4.5)
≥ 40 years	5,511 (0.36)	236 (0.56)	-	-	193 (0.47)	23 (0.35)	-	157 (0.52)	218 (0.07)	2,002 (0.31)	413 (0.19)	2,269 (0.89)
No children	187,425 (12.1)	6,542 (15.5)	329 (5.2)	295 (5.8)	15,748 (38.1)	3,244 (50.1)	255 (7.7)	3,739 (12.4)	3,956 (1.3)	72,831 (11.4)	32,816 (15.2)	47,670 (18.7)
Unknown	35,811 (2.3)	3,312 (7.8)	6,043 (94.8)	4,770 (94.2)	390 (0.94)	168 (2.6)	3074 (92.3)	3,148 (10.4)	148 (0.05)	11,709 (1.8)	2,332 (1.1)	717 (0.28)
Menopausal status												
Pre-menopausal	244,562 (15.8)	9,689 (23.0)	4,018 (63.1)	2,941 (58.1)	24,718 (59.8)	4,754 (73.4)	1232 (37.0)	11,796 (39.1)	128,741 (42.8)	1 (0.00)	0 (0.00)	56,672 (22.2)
Post-menopausal	1,301,655 (84.2)	32,505 (77.0)	2,354 (36.9)	2,124 (41.9)	16,617 (40.2)	1,726 (26.6)	2097 (63.0)	18,352 (60.9)	172,168 (57.2)	639,025 (100.0)	215,905 (100)	198,782 (77.8)
Age at menopause (postmenopausal women only)												
< 40 years	77,087 (5.9)	2,594 (8.0)	406 (17.2)	292 (13.7)	0 (0.00)	-	131 (6.2)	2,004 (10.9)	6,464 (3.8)	21,158 (3.3)	38,407 (17.8)	5,631 (2.8)
40–44 years	124,498 (9.6)	3,005 (9.2)	639 (27.1)	518 (24.4)	1,508 (9.1)	-	251 (12.0)	2,008 (10.9)	18,184 (10.6)	52,184 (8.2)	33,393 (15.5)	12,808 (6.4)
45–49 years	296,861 (22.8)	5,383 (16.6)	757 (32.2)	653 (30.7)	3,142 (18.9)	-	556 (26.5)	3,953 (21.5)	65,017 (37.8)	132,074 (20.7)	51,357 (23.8)	33,969 (17.1)
50–54 years	438,854 (33.7)	7,762 (23.9)	340 (14.4)	375 (17.7)	4,893 (29.4)	-	964 (46.0)	6,294 (34.3)	60,141 (34.9)	223,470 (35.0)	66,050 (30.6)	68,565 (34.5)
≥ 55 years	104,681 (8.0)	12,232 (37.6)	103 (4.4)	73 (3.4)	739 (4.4)	-	173 (8.2)	1,051 (5.7)	7,779 (4.5)	46,200 (7.2)	14,617 (6.8)	21,714 (10.9)
Unknown	259,674 (19.9)	1,529 (4.7)	109 (4.6)	213 (10.0)	6,335 (38.1)	1,726 (100)	22 (1.0)	3,042 (16.6)	14,583 (8.5)	163,939 (25.7)	12,081 (5.6)	56,095 (28.2)
Ever used oral contraceptives												
Yes	791,983 (51.3)	24,802 (58.8)	223 (3.5)	349 (6.9)	30,475 (73.7)	3,582 (55.3)	523 (15.7)	20,167 (66.9)	29,611 (9.8)	391,488 (61.3)	84,234 (39.0)	207,052 (81.1)
No	742,243 (48.1)	17,392 (41.2)	6,148 (96.5)	4,688 (92.6)	10,503 (25.4)	2,848 (44.0)	2778 (83.4)	9,521 (31.6)	271,252 (90.1)	243,805 (38.2)	128,537 (59.5)	47,549 (18.6)
Unknown	8,662 (0.6)	-	1 (0.02)	28 (0.55)	357 (0.86)	50 (0.77)	28 (0.01)	460 (1.5)	46 (0.02)	3,733 (0.58)	3,134 (1.45)	853 (0.33)
Ever used hormone replacement therapy												
Yes	584,677 (37.8)	16,619 (39.4)	-	-	7,506 (18.2)	-	654 (19.6)	8,198 (27.2)	-	341,137 (53.4)	114,186 (52.9)	96,377 (37.7)
No	628,851 (40.7)	24,294 (57.6)	-	-	33,239 (80.4)	-	1432 (43.0)	20,997 (69.6)	-	289,096 (45.2)	101,719 (47.1)	158,074 (61.9)
Unknown	332,689 (21.5)	1,281 (3.0)	6,372 (100)	5,065 (100)	590 (1.4)	6,480 (100)	1243 (37.3)	953 (3.2)	300,909 (100)	8,793 (1.4)	0 (0.00)	1,003 (0.39)

Abbreviations: AHS-2, Adventist Health Study-2; CARRS, Centre for cArdiometabolic Risk Reduction in South Asia; CKB, China Kadoorie Biobank; EPIC, European Prospective Investigation into Cancer and Nutrition; MWS, Million Women Study; NIH-AARP, National Institutes of Health-AARP Diet and Health Study; OVS, Oxford Vegetarian Study; TCHS, Tzu Chi Health Study; UKWCS, UK Women’s Cohort Study.

^1^All values are N (%).

^2^Sex-specific tertiles of metabolic equivalents.

c indicates that no information was available for this variable in the specified cohort.

The original article [1] has been corrected.

